# High-throughput neutralization assay for multiple flaviviruses based on single-round infectious particles using dengue virus type 1 reporter replicon

**DOI:** 10.1038/s41598-018-34865-y

**Published:** 2018-11-09

**Authors:** Mami Matsuda, Atsushi Yamanaka, Keigo Yato, Kentaro Yoshii, Koichi Watashi, Hideki Aizaki, Eiji Konishi, Tomohiko Takasaki, Takanobu Kato, Masamichi Muramatsu, Takaji Wakita, Ryosuke Suzuki

**Affiliations:** 1 Department of Virology II, National Institute of Infectious Diseases, 1-23-1, Toyama, Shinjuku-ku, Tokyo 162-8640 Japan; 2 BIKEN Endowed Department of Dengue Vaccine Development Faculty of Tropical Medicine, Mahidol University, 420/6 Ratchawithi Road, Ratchahewi, Bangkok 10440 Thailand; 3 BIKEN Endowed Department of Dengue Vaccine Development, Research Institute for Microbial Diseases, Osaka University, 3-1 Yamadaoka, Suita, Osaka 565-0871 Japan; 4 Laboratory of Public Health, Faculty of Veterinary Medicine, Hokkaido University, Kita 18, Nishi 9, Kita-ku, Sapporo, 060-0818 Japan; 5 Kanagawa Prefectural Institute of Public Health, 1-3-1, Shimomachiya, Chigasaki, Kanagawa 253-0087 Japan; 6 Department of Virology II, National Institute of Infectious Diseases, 4-7-1 Gakuen, Musashi-murayama-shi, Tokyo, 208-0011 Japan

## Abstract

Diseases caused by the genus *Flavivirus*, including dengue virus (DENV) and Zika virus (ZIKV), have a serious impact on public health worldwide. Due to serological cross-reactivity among flaviviruses, current enzyme-linked immunosorbent assay (ELISA) for IgM/G cannot reliably distinguish between infection by different flaviviruses. In this study, we developed a reporter-based neutralization assay using single-round infectious particles (SRIPs) derived from representative flaviviruses. SRIPs were generated by transfection of human embryonic kidney 293 T cells with a plasmid encoding premembrane and envelope (prME) proteins from DENV1–4, ZIKV, Japanese encephalitis virus, West Nile virus, yellow fever virus, Usutu virus, and tick-borne encephalitis virus, along with a plasmid carrying DENV1 replicon containing the luciferase gene and plasmid for expression of DENV1 capsid. Luciferase activity of SRIPs-infected cells was well correlated with number of infected cells, and each reporter SRIP was specifically neutralized by sera from mice immunized with each flavivirus antigen. Our high-throughput reporter SRIP-based neutralization assay for multiple flaviviruses is a faster, safer, and less laborious diagnostic method than the conventional plaque reduction neutralization test to screen the cause of primary flavivirus infection. The assay may also contribute to the evaluation of vaccine efficacy and assist in routine surveillance and outbreak response to flaviviruses.

## Introduction

Many flaviviruses cause significant human morbidity and mortality, including dengue virus (DENV), Zika virus (ZIKV), yellow fever virus (YFV), West Nile virus (WNV), Japanese encephalitis virus (JEV), and tick-borne encephalitis virus (TBEV), and are transmitted by mosquitoes or ticks. Flavivirus is an enveloped, positive-sense RNA virus with a genome of about 11 kb. A single open reading frame encodes the viral polyprotein, which is processed into structural [capsid (C), premembrane/membrane (prM/M), and envelope (E)] and nonstructural (NS1, NS2A, NS2B, NS3, NS4A, NS4B, and NS5) proteins by viral and host proteases. This open reading frame is flanked by 5′ and 3′ untranslated regions. Viral structural proteins form infectious viral particles, while nonstructural proteins are responsible for various functions, including viral replication and evasion of host immune response^1^.

Various human pathogenic flaviviruses have become more widespread recently^2,3^. Therefore, methods to specifically diagnose infections caused by these flaviviruses are needed. However, laboratory-confirmed diagnosis of specific flavivirus infections remains complex^4–6^ because the duration of viremia in flavivirus infection is short and may be at low or undetectable levels. Therefore, a negative result by RT-PCR, the most common assay due to its sensitivity and specificity, in a patient sample does not exclude the possibility of flavivirus infection. Accordingly, serological testing is important for diagnosis of such infection. However, it is also known that IgM-capture ELISA, the most commonly used assay for serological testing, can yield false-positive results because of cross-reacting antibodies against related flaviviruses or nonspecific reactivity^7–9^. The plaque reduction neutralization test (PRNT), which is a more specific and sensitive test than ELISA, is currently recommended as the gold standard serological test. Unfortunately, cross-neutralization by antibodies against related flaviviruses is still partially observed. As a result, a comparison of the endpoint titers obtained using multiple flaviviruses should be performed^10,11^, although the standard PRNT is not suitable for high-throughput processing because it is time-consuming and labor-intensive. In addition, a BSL3 facility is required to perform PRNT if live WNV or TBEV is used.

We previously reported that single-round infectious particles (SRIPs) for JEV, DENV, and ZIKV can be successfully used in place of authentic viruses in neutralization tests with an advantage in terms of safety^12–14^ because SRIP-infected cells are not able to produce progeny virions due to the lack of partial structural gene in the packaged genome. In this study, we demonstrate that reporter SRIPs from several flaviviruses can be used as viral particles for neutralization assays as a faster and safe diagnostic method of flavivirus infection.

## Results

### Characterization of DENV1 replicon encoding the luciferase gene

**Figure 1:**
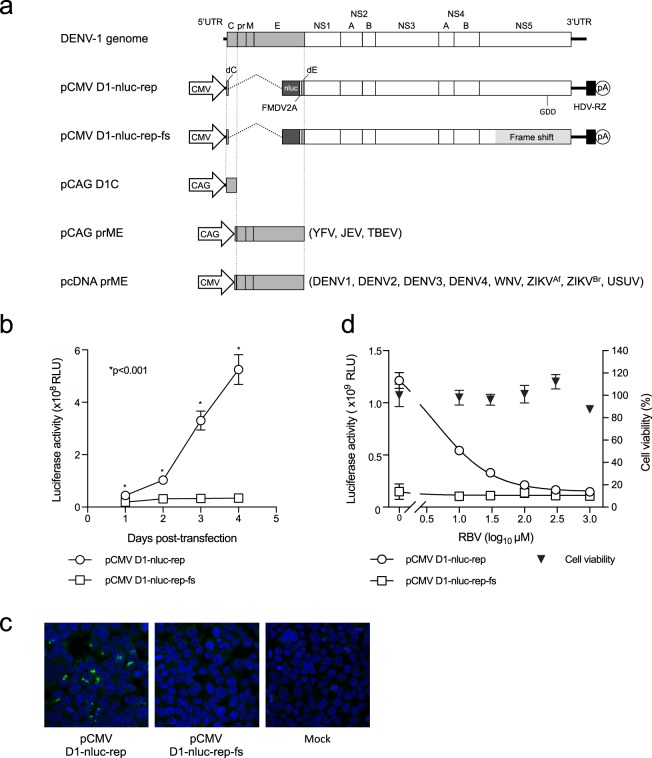
Construction and characterization of a DNA-based DENV1 replicon containing the NanoLuc gene. (**a**) Schematic representation of the DENV1 genome and replicon construct showing the position of the CMV promoter (CMV), NanoLuc gene (nluc), 2 A protein sequence of foot-and-mouth disease virus (FMDV2A), hepatitis delta virus ribozyme (HDV-RZ), and polyadenylation signal (pA). Structural protein-expression plasmids used to generate SRIPs are also shown. (**b**) Expression of reporter genes in DENV1 replicon. 293 T cells were transfected with pCMV-D1-nluc-rep or pCMV-D1-nluc-rep-fs, and luciferase activity was monitored at indicated time points. The mean and standard deviation calculated from triplicate NanoLuc values for each replicon are presented in the graph. The statistical significance of differences was evaluated using Student’s t-test. (**c**) dsRNA staining of cells transfected with replicon plasmid. 293T cells were transfected with the indicated plasmids and then stained with anti-dsRNA antibody (Green). Cell nuclei were counterstained with DAPI. (**d**) Luciferase activity of 293T cells transfected with pCMV-D1-nluc-rep in the presence of ribavirin at the indicated concentration. Detection was performed at 3 days post-transfection. Data are expressed as means of triplicate values with error bars indicating standard deviations.

In this study, plasmid-based subgenomic DENV1 replicon containing the nanoluciferase (NanoLuc) gene (pCMV-D1-nluc-rep) was generated as shown in Fig. 1. pCMV-D1-nluc-rep-fs, which contains a frameshift mutation upstream of the GDD motif of RNA-dependent RNA polymerase, was also constructed as a negative control. In order to characterize the replication property of the plasmid-derived replicon, 293 T cells were transfected with the generated plasmids. Cells transfected with pCMV-D1-nluc-rep had significantly increased luciferase activity in a time-dependent manner compared with that of pCMV-D1-nluc-rep-fs (Fig. 1b). Indirect immunofluorescence with an anti-dsRNA antibody showed positive staining in the cytoplasm of cells transfected with pCMV-D1-nluc-rep, whereas no immunofluorescent signal was detected in cells transfected with pCMV-D1-nluc-rep-fs (Fig. 1c). Furthermore, luciferase activity of cells transfected with pCMV-D1-nluc-rep decreased by treatment with ribavirin, a broad-spectrum antiviral agent, in a dose-dependent manner without cytotoxicity (Fig. 1d). These results indicate that subgenomic replicon RNAs transcribed intracellularly from plasmid pCMV-D1-nluc-rep replicate in the cells, and replication levels can be monitored by luciferase activity.

### Generation of various flavivirus SRIPs with DENV1 reporter replicon

**Figure 2:**
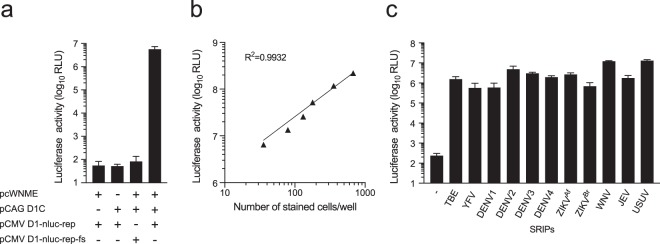
Generation of multiple flavivirus SRIPs with different prME plasmids. (**a**) Luciferase activity of SRIPs produced by transfection of 293T cells with replicon plasmid and structural protein-expression plasmids. Each plasmid used for transfection is indicated. The supernatants of transfected cells collected at 3 days post-transfection were used to inoculate Vero cell monolayers. Luciferase activity of infected cells was subsequently determined at 3 days post-infection. (**b**) Relationship between luciferase activity and a number of infected cells. WNV-SRIPs were serially diluted (2-fold dilutions) and used to inoculate Vero cells. Luciferase activity and a number of cells stained with anti-NS1 antibody were plotted. The dotted line indicates the linear regression line. The coefficient of determination (R^2^) is displayed in the graph. (**c**) Luciferase activity of SRIPs produced by transfection of 293T cells with replicon plasmid and structural protein-expression plasmids. Each prME plasmid used for transfection is indicated. The supernatants of transfected cells collected at 3 days post-transfection were used to inoculate Vero cell monolayers. Luciferase activity of cells was subsequently determined at 3 days post-infection.

In order to confirm the production of flavivirus reporter SRIPs, 293T cells were transfected with a mixture of three plasmids (pCMV-D1-nluc-rep, pCAG-D1C, and WNV prME-expression plasmids). Then, Vero cells were inoculated with the culture supernatant of transfected cells and luciferase activity was measured. Vero cells inoculated with the supernatant of cells transfected with the three plasmids showed high luciferase activity (Fig. 2a). In contrast, Vero cells inoculated with the supernatant of cells transfected with plasmids without pCAG-D1C or WNV prME-expression plasmids, or combination of pCMV-D1-nluc-rep-fs, pCAG-D1C, and prME-expression plasmids, showed lower luciferase activity. These data suggest that infectious WNV-SRIPs can be produced by transfection of 293T cells with pCMV-D1-nluc-rep, pCAG-D1C, and prME-expression plasmids.

Next, to examine the correlation between the number of infected cells and luciferase activity of cells infected with reporter SRIPs, WNV-SRIPs were serially diluted and used to infect Vero cells, followed by immunostaining and luciferase activity measurement. As shown in Fig. 2b, luciferase activity levels in SRIPs-infected cells were well correlated with the number of infected cells, indicating that the luciferase assay can be used to quantify viral infection in reporter SRIP-infected cells. Furthermore, in order to observe inter-well and inter-experiment variation, equal amounts of WNV-SRIPs were used to inoculate Vero cells and luciferase activity was measured. The mean signal and %CV were calculated for the same plate (inter-well %CV) and three independent experiments (inter-experiment %CV). Low variation with a mean inter-well CV of less than 16.7% and inter-experiment CV of less than 18.1% was observed, indicating acceptable assay performance. Different infectious titers of WNV-SRIPs were also applied to calculate Z-factor, as shown in Supplementary Figure 1. The average Z-factor value was above 0.5, showing the robustness of the assay.

We also tested whether other flavivirus reporter SRIPs could be produced by using the same procedure with prME-expression plasmids for the following viruses: TBEV, YFV, DENV1, DENV2, DENV3, DENV4, two strains of ZIKV, JEV, and Usutu virus (USUV). All reporter SRIPs examined showed significantly high luciferase activity compared with negative control (without prME-expression plasmid) (Fig. 2c), suggesting that pCMV-D1-nluc-rep can be used to produce multiple flavivirus SRIPs.

### Comparison of ZIKV-SRIP and ZIKV in a neutralization test

**Figure 3:**
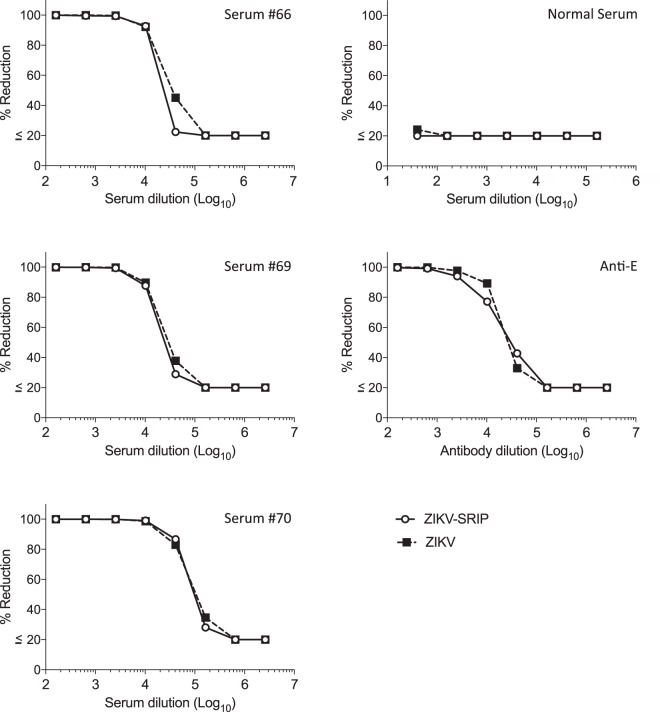
Comparison of ZIKV-SRIP and ZIKV in a neutralization test using three Zika NS1 IgG-positive human serum (#66, #69, and #70), one serum from normal healthy donor, and anti-E monoclonal antibody. Serum samples were serially four-fold diluted and incubated with ZIKV-SRIP or authentic ZIKV overnight at 4 °C. The mixture was then titrated on Vero cells. Dose-dependent percentage reduction curves were obtained with ZIKV-SRIP (open circles) and ZIKV virus (closed square).

One of the flavivirus SRIPs (ZIKV-SRIPs) was further evaluated by comparing it with authentic live virus using three Zika NS1 IgG-positive human serum, one serum from Japanese normal healthy donor, and an anti-E monoclonal antibody (Fig. 3). The dose-response curves obtained using either ZIKV-SRIP or authentic ZIKV were similar in the neutralization tests using all tested serum or antibody. This result indicates that the ZIKV-SRIP antigen can be successfully used in place of authentic ZIKV antigen in Vero cell neutralization tests in accordance with previous reports^14^.

### Neutralization test with sera from mice immunized with monovalent prME plasmid

**Figure 4:**
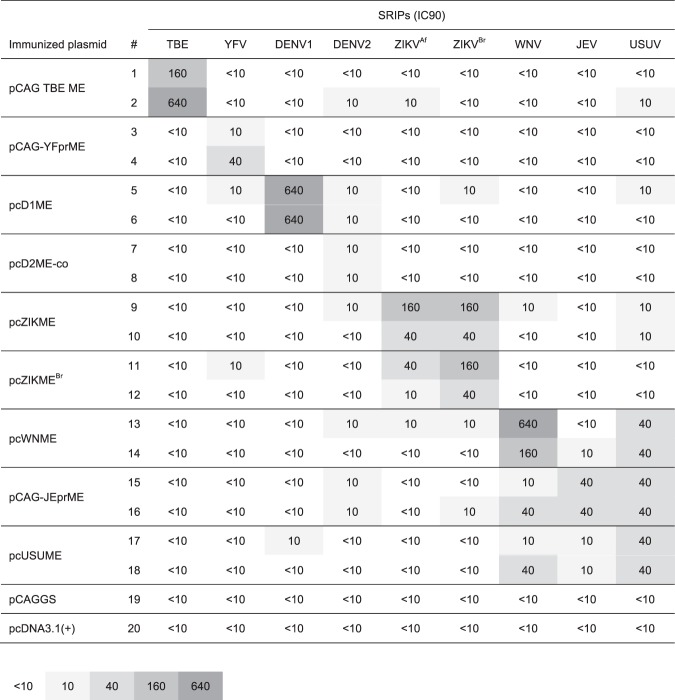
Neutralization testing of sera from mice immunized with each plasmid expressing flavivirus prME. BALB/c mice were immunized with the indicated plasmids. Mice sera were subjected to neutralization test using indicated SRIPs. The neutralization titers were shown as inhibitory concentration that neutralized 90% of SRIP infection (IC90). TBEV, tick-borne encephalitis virus; YFV, yellow fever virus; DENV, dengue virus; ZIKV, Zika virus; JEV, Japanese encephalitis virus; WNV, West Nile virus; USUV, Usutu virus; NT, not tested.

To evaluate the specificity of each flavivirus reporter SRIP, we conducted neutralization tests with sera from mice immunized with each prME-expression plasmid (Fig. 4). Almost all immunized mice showed the highest neutralizing antibody titers against homologous flavivirus SRIPs, although weak cross-neutralization against heterologous flaviviruses was also partially observed. These results suggest that reporter SRIPs can be neutralized by specific antibody and used as viral particles for the neutralization assay. It should be noted that sera of mice immunized with pCAG-JEprME neutralized USUV-SRIPs to the same levels as with JEV-SRIPs, and one of two mice (#16) neutralized WNV-SRIPs at the same levels as with JEV-SRIPs. In addition, sera of one of the two mice (#18) immunized with pcUSUME neutralized WNV-SRIPs at the same levels as with USUV-SRIPs.

## Discussion

In this study, we demonstrate the utility of multiple flavivirus reporter SRIPs as an alternative tool to authentic flaviviruses in neutralization assays. Large-scale reporter SRIPs can be easily produced by co-transfection with plasmids, DENV1 replicon plasmid, DENV1 capsid expression plasmid, and prME-expression plasmid. By using nucleotide sequence information of prME, various flavivirus SRIPs can be readily produced as compared with production of an infectious clone or even importation of the infectious virus itself from different countries. It has been shown that dose-dependent neutralizing and enhancing antibody activity curves obtained using SRIPs and viruses were equivalent in DENV1–4 and ZIKV^13,14^. Here, introduction of a reporter gene into the DENV1 replicon enabled creation of a high-throughput neutralization assay (96-well plate format), which is important for large-scale processing as well as handling samples with limited volumes.

A reliable high-throughput assay to detect flavivirus neutralization antibodies is critical for laboratory and clinical studies of flavivirus infection. Recently, novel methods for neutralizing assays have been described using Dengue or Zika viruses containing the luciferase reporter gene^15–17^. These reporter virus-based assays for neutralizing antibody evaluation showed a strong linear correlation with conventional plaque-based assays. In addition, the reporter virus-based assay significantly improved throughput to allow testing of a large number of samples. However, these reporter flaviviruses are infectious; therefore, proper biosafety practices are required. In addition, construction of reporter flavivirus is thus far limited to only some viruses of specific strains. The fluorescent neutralization test is a fast and robust assay that offers significant advantages over classical PRNT^18^, however, live virus is still required. Conversely, the SRIP-based neutralization assay has advantages concerning the construction of the different flaviviruses, since only the prME sequence is needed. This advantage prompted us to easily and rapidly produce 11 different flavivirus SRIPs in this study, however, some SRIPs showed lower infectious titer compared to live viruses. In addition, a BSL3 facility is not required for the SRIP-based assay when live viruses including reporter viruses are used (e.g., YFV, WNV, and TBEV).

We applied reporter SRIPs in a neutralization assay to evaluate sera of mice immunized with different flavivirus prME plasmids and human sera. The neutralization assay with sera from mice immunized with each prME plasmid revealed that almost all immunized mice showed the highest neutralizing antibody titers against homologous flavivirus SRIPs (Fig. 4). However, sera of mice immunized with prME of the JEV serogroup (JEV, WNV, and USUV) showed substantial cross-reactivity against SRIPs belonging to the same serogroup. In particular, sera of mice immunized with pCAG-JEprME neutralized USUV-SRIPs at the same levels as with JEV-SRIPs. These results can be explained by the high amino acid sequence similarity^19^ and close antigenic property between these two viruses. In fact, E protein of JEV, USUV, and WNV exhibited more than 90% similarity at the amino acid level. These results raised the concern of the difficulty in differentiating infection of these two viruses by neutralization assays. However, we could not exclude the possibility that neutralizing titers against different viruses of JEV serogroup are the result of a substantial low neutralizing titer by immunization in mice with DNA compared to that of a virus-infected human that had infected with the virus. Thus, differentiation of neutralization titers among the JEV serogroup should be further examined in detail using sera from patients infected with each virus.

Sera of mice immunized with pcZIKME (MR766-NIID; African strain) showed similar neutralizing titers against ZIKV^Af^-SRIPs (MR766-NIID; African strain) and ZIKA^Br^-SRIPs (SHP2015; Asian strain). In contrast, sera of mice immunized with pcZIKME^Br^ (SHP2015; Asian strain) showed 4-fold higher titers against homologous ZIKA^Br^-SRIPs than against heterologous ZIKV^Af^-SRIPs. These data are consistent with previous reports of mice immunized with different strains of ZIKV^20,21^ and suggested that a neutralization assay using multiple ZIKV strains for detection of serological differences between the ancestral African strain and Asian strain might be useful to determine precisely ZIKV infection. It will be of interest to explore these associations with various ZIKV strains.

In conclusion, our high-throughput reporter SRIP-based neutralization assay for multiple flaviviruses is a faster and safer method than the conventional PRNT assay to screen the cause of primary flavivirus infection. This assay could be applied for other flaviviruses with prME sequences, contribute to the evaluation of vaccine efficacy, and assist in the routine surveillance and outbreak response to flaviviruses as well as basic research of flaviviruses virology.

## Methods

### Plasmid construction

We used the D1/Hu/Saitama/NIID100/2014 strain, isolated from a female with Dengue fever in Yoyogi, Tokyo, Japan in 2014^22^, to construct the DENV1 subgenomic replicon plasmid containing the luciferase gene. Viral RNA was extracted from infected Vero cells, reverse transcribed into cDNA, and amplified in individual dsDNA fragments containing cytomegalovirus (CMV) promoter, nanoluciferase (NanoLuc) gene following foot-and-mouth disease virus (FMDV) 2A gene, and hepatitis delta virus ribozyme (HDV-RZ), as shown in Fig. 1a. The NanoLuc gene was introduced at the position 17 aa after the C-coding region, following the FMDV-2A gene, and 24 aa before the C-terminal transmembrane domain of the E protein coding sequence. Several individual fragments required to produce a replicon-length cDNA were readily assembled into the low copy number plasmid pACYC177, designated pCMV-D1-nluc-rep. Sequence analyses confirmed that there was only 1 nucleotide difference in NS4B from the deposited sequence of D1/Hu/Saitama/NIID100/2014, resulting in an amino acid change at position 120 (Ala to Thr).

pCMV-D1-nluc-rep-fs, which contains a frameshift mutation through a 4-nt insertion upstream of the GDD motif of RNA-dependent RNA polymerase in NS5, was also constructed and used as a negative control with no replication activity.

To generate the DENV1 capsid expression plasmid pCAG-D1C, cDNA encoding the mature capsid consisting of 100 aa was amplified from cDNA used for amplification of the replicon. The resultant fragments were cloned into pCAGGS.

To generate the DENV1 NS1 expression plasmid, cDNAs encoding the C-terminal 29 aa of E and entire NS1 with a FLAG tag at the C terminus were amplified by PCR. The resultant fragments were cloned into pCAGGS.

Plasmids pcD1ME, pCAG-YFprME, pCAG-JEprME, pcWNME, pcZIKME, pcUSUME, and pCAG-TBEME are expression plasmids for the signal, prM, and E of DENV1 (Mochizuki), YFV (17D-204), JEV (Nakayama), WNV (NY99-6922), ZIKV (MR766-NIID), USUV (NC_006551), and TBEV (Oshima 5–10), respectively, as described previously^12,14^.

Other pcDNA3-based expression plasmids encoding prM and E of DENV2 (pcD2ME^co^), DENV3 (pcD3ME^SG^), DENV4 (pcD4ME^Th^), and ZIKV^Br^ (pcZIKME^Br^) were constructed from synthetic DNA by GENEWIZ Japan (Tokyo, Japan) based on nucleotide sequence information available in GenBank (accession numbers: JX042506 for DENV2, EU081222 for DENV3, AY618988 for DENV4, and KU321639 for ZIKV^Br^).

### Cells and Reagents

Human embryonic kidney 293T cells and African green monkey kidney Vero cells were maintained in Dulbecco’s modified Eagle’s medium (Wako Pure Chemical Industries) supplemented with non-essential amino acids, penicillin (100 U/ml), streptomycin (100 mg/ml), and 10% fetal bovine serum (FBS). All cell lines were cultured at 37 °C in a 5% CO_2_ incubator. Ribavirin was purchased from Sigma-Aldrich.

### Cell viability assay

Cell viability was determined by the CellTiter-Glo Luminescent Cell Viability Assay (Promega).

### Production of each SRIP

293T cells were grown in a 10-cm dish and co-transfected with three plasmids: 2.5 µg of replicon plasmid, 1.25 µg of capsid-expression plasmid, and 1.25 µg of each prME-expression plasmid, using polyethylenimine. Culture medium was removed and replaced with fresh medium supplemented with 10 mM HEPES buffer at 2 days post-transfection. The medium was harvested after 3 days post-transfection and used as SRIPs. Infectious titer of generated SRIPs was estimated by infection in Vero cells with subsequent luciferase assay.

### Immunization of mice

Specific pathogen-free female BALB/c mice (9 weeks old) were purchased from SLC Japan and immunized with a plasmid that encoded each prME or empty vector. Then, 50 U of hyaluronidase were injected into the quadriceps muscles. After 15 min, mice were anesthetized with isoflurane and injected with 20 μg of each plasmid in the quadriceps muscles. The same procedure was performed on the other quadriceps muscle. Electrode needles were then inserted into the muscle and electric pulses were delivered using an electric pulse generator (NEPA21, Nepa Gene). Three poring pulses (50 V, 30 ms) followed by three transfer pulses (20 V, 50 ms) were administered to each injection site three times. A booster immunization was given 2 and 4 weeks after the primary immunization. Sera were collected 3 weeks after the last immunization. Blood samples were collected in a Bloodsepar (Immuno-Biological Laboratories) and centrifuged at 2500 × g for 2 min at room temperature. The supernatants were collected as sera and heat-inactivated at 56 °C for 30 min for use in neutralization assays. All animal experiments were approved by the Animal Care and Use Committee of the National Institute of Infectious Diseases and carried out in accordance with the approved guidelines.

### Antibody

Antibodies directed against DENV1-NS1 were raised by genetic immunization of BALB/c mice with a DENV1-NS1 expression plasmid. In brief, animals received five applications of 40 μg (20 μg/shot) of the plasmid in the quadriceps muscles using the electric pulse generator NEPA21. Anti-dsRNA antibody (J2) was obtained from English & Scientific Consulting Kft. Anti-Flavivirus E mouse monoclonal antibody (D1-4G2-4-15) was obtained from Merck.

### Immunostaining

To determine the number of SRIP-infected cells, Vero cells were plated in multiwell plates and incubated with diluted samples. Following 3-day incubation, monolayers were rinsed with phosphate-buffered saline (PBS), fixed in cold acetone/methanol (1:1), and then blocked with a non-fat milk solution (Block Ace; Snow Brand Milk Products) for 30 min at room temperature. Samples were then incubated with mouse anti-DENV1 NS1 serum for 60 min at room temperature, followed by Alexa Fluor 488-labeled goat anti-mouse IgG secondary antibody (Invitrogen).

For staining dsRNA, 293T cells were transfected with the indicated plasmids. Four days post-transfection, cells were fixed and permeabilized. Samples were then incubated with anti-dsRNA antibody. Green signals were obtained with Alexa-Fluor-488-labelled goat anti-mouse IgG secondary antibody (Invitrogen). Cell nuclei were counterstained with DAPI.

### Neutralization tests

SRIPs (50–100 infectious units/well) were used for the neutralization assay. Serial dilutions of serum samples (serial 4-fold dilutions) were mixed with SRIPs at a 1:1 ratio. After incubating the mixtures on ice overnight, each mixture was added to 96-well tissue culture plates containing Vero cell monolayers. After incubating the plate at 37 °C for 5–6 h, media were changed, and the plate was further incubated at 37 °C. Luciferase activity of cells was subsequently determined at 3 days post-infection using the Nano-Glo Luciferase Assay System (Promega). The neutralization titer was determined as the serum dilution that inhibited more than 90% of the SRIP inoculum without serum (IC90).

MR766-NIID strain of ZIKV (50–100 focus-forming units/well) was used for the neutralization assay. Serial dilutions of samples were mixed with the virus at a 1:1 ratio. After incubating the mixtures on ice overnight, each mixture was added to 96-well tissue culture plates containing Vero cell monolayers. After incubating the plate at 37 °C for 5–6 h, the supernatants then were replaced with fresh medium containing 10% FBS and 0.8% carboxymethyl cellulose. Following incubation for 30 hours at 37 °C, the monolayers were fixed and immunostained with the anti-E antibody followed by an Alexa Fluor 488-conjugated anti-mouse secondary antibody (Invitrogen). Stained foci were counted and used to calculate the percent reduction of infection.

### Serum samples

Zika NS1 IgG-positive human serum samples were purchased from TRINA BIOREACTIVES AG. Sera from Japanese healthy donor was purchased from Clinical Trials Laboratory Services.

## Electronic supplementary material

Supplementary Figure 1
