# Supplementary material for: High-throughput neutralization assay for multiple flaviviruses based on single-round infectious particles using dengue virus type 1 reporter replicon
Source: Sci Rep. 2018 Nov 9;8:16624. doi: 10.1038/s41598-018-34865-y (PMC6226426; doi:10.1038/s41598-018-34865-y)
Supplement: Supplementary file 1 — Supplementary Figure 1 [file 41598_2018_34865_MOESM1_ESM.pdf]

High-throughput neutralization assay for multiple flaviviruses based on single-round infectious particles using dengue virus type 1 reporter replicon

Mami Matsuda[1], Atsushi Yamanaka[2,3], Keigo Yato[1], Kentaro Yoshii[4], Koichi Watashi[1], Hideki Aizaki[1], Eiji Konishi[2,3], Tomohiko Takasaki[5], Takanobu Kato[1], Masamichi Muramatsu[1], Takaji Wakita[1], Ryosuke Suzuki[1]*

[1] Department of Virology II, National Institute of Infectious Diseases, 1-23-1, Toyama, Shinjuku-ku, Tokyo 162-8640, Japan

[2] BIKEN Endowed Department of Dengue Vaccine Development Faculty of Tropical Medicine, Mahidol University, 420/6 Ratchawithi Road, Ratchahewi, Bangkok 10440, Thailand

[3] BIKEN Endowed Department of Dengue Vaccine Development, Research Institute for Microbial Diseases, Osaka University, 3-1 Yamadaoka, Suita, Osaka 565-0871, Japan

[4] Laboratory of Public Health, Faculty of Veterinary Medicine, Hokkaido University, Kita 18, Nishi 9, Kita-ku, Sapporo 060-0818, Japan

[5] Kanagawa Prefectural Institute of Public Health, 1-3-1, Shimomachiya, Chigasaki, Kanagawa 253–0087, Japan

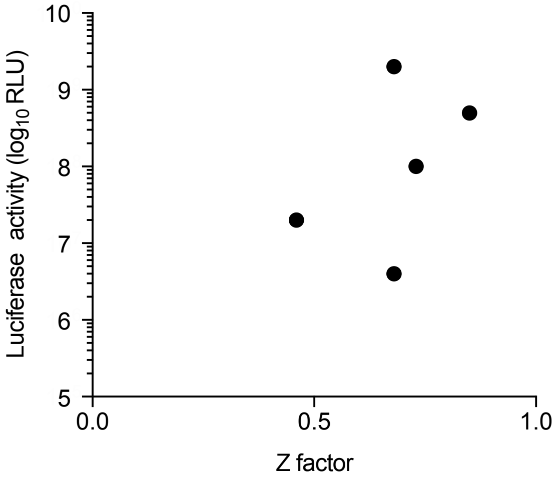

Supplementary Figure 1. Different infectious titers of WNV-SRIPs were inoculated with Vero cells, and luciferase activity of cells with or without infection was subsequently determined at 3 days post-infection. Luciferase activity and Z-factor were plotted.
